# Progesterone Regulation of Milk Fat Globule Size Is VLDL Dependent

**DOI:** 10.3389/fendo.2020.00596

**Published:** 2020-09-09

**Authors:** Nurit Argov-Argaman, Chen Raz, Zvi Roth

**Affiliations:** Department of Animal Science, The Robert H. Smith Faculty of Agriculture, Food and Environment, The Hebrew University of Jerusalem, Jerusalem, Israel

**Keywords:** mammary epithelial cells, milk, fat globule, estrous, progesterone, VLDL

## Abstract

Progesterone plays a pivotal role during mammogenesis and serves as an inhibitor of the secretory activation of mammary cells in the last days of gestation. However, its role during lactogenesis, in particular its involvement in lipid metabolism, and milk fat content and composition, is unknown. Here, we provide new evidence of progesterone's involvement in the regulation of milk fat globule (MFG) synthesis and secretion. Findings from both *in vivo* and *in vitro* studies indicated that the concentration and the direction (increase vs. decrease) of progesterone concentration to which the mammary epithelial cells (MECs) are exposed affect MFG size. This was found to be very-low-density lipoprotein (VLDL) dependent: in the presence of VLDL, the proportion of MEC with small lipid droplets (<1 μm) increased 2.4-fold, and the proportion of large lipid droplets (>1 μm) increased 4-fold; in the absence of VLDL, no differences were found. The findings add to our understanding of the mechanism underlying the regulation of MFG size and provide new evidence for progesterone's role in lipid metabolism in the mammary gland during lactogenesis. The fact that the size, synthesis, and composition of MFG are affected by the cyclic pattern of progesterone concentration in the circulation might have physiologically relevant consequences, in particular on milk as a nutritional source.

## Introduction

The role of progesterone in mammogenesis (1) and secretory activation of mammary cells in the last days of gestation has been thoroughly studied and well-documented (2). However, the role of progesterone during lactation is not well-described. Nevertheless, there are some evidences that progesterone is involved in lipid metabolism pathways in the mammary gland. This process include synthesis, secretion, and size regulation of milk fat globules (MFGs), regulation of total fat content in the cell, as well as the stability of its membranes [reviewed by Argov-Argaman (3)]. Whether progesterone is involved in the regulation of MFG synthesis and secretion has never been studied. Moreover, progesterone's possible role in regulating MFG composition and structure has not been documented.

The MFG is the structure utilized by the mammary gland epithelial cells (MECs) to secrete milk fat. Formation of the MFG initiates in the endoplasmic reticulum through the release of a triglyceride (Tg) droplet covered with one layer of endoplasmic reticulum phospholipids (PL) into the cytoplasm. From its origin site, the cytoplasmic lipid droplet migrates to the apical pole of the cell, where it is pinocytosed into the alveolar lumen. During this process, it is enveloped by the plasma membrane bilayer, forming the MFG membrane (4). The MFG membrane consists of glycoproteins (20–60%) and glycerophospholipids (33%), primarily phosphatidylcholine (PC), and sphingomyelin (SM), and more minor contents of phosphatidylethanolamine (PE), phosphatidylinositol (PI), and phosphatidylserine (PS) (5).

The MFGs are secreted into milk in a wide range of sizes, from 200 nm to 15 μm (6, 7). The average diameter is associated with lactation stage (6), energy balance (5), diet (8, 9), genetic background (8, 10), and interactions among these factors (11). The differences in MFG size may be attributed to several mechanisms including, but not limited to, fusion between intracellular lipid droplets during their migration to the apical pole of the cell (12–14); the balance between lipolysis and lipogenesis processes, regulated by lipases and PAT proteins, which in turn regulate Tg hydrolysis in the lipid droplet (15); and limited availability of envelope material such as PC, which results in a higher volume-to-surface area ratio and increased lipid droplet size (16, 17). The availability of long-chain fatty acids has been suggested to limit the capacity to synthesize membrane material (18), consequently also affecting lipid droplet size (16, 17). This hypothesis is further supported by our recent findings indicating different amounts and sizes of lipid droplet in MECs treated with fatty acids at different levels of bioavailability (19). Whether this process is also regulated by progesterone has not been documented.

Many of the lipogenic processes are regulated by the animal's metabolic status (15). Some studies have demonstrated a role for progesterone. For instance, in the mammary gland tumor cell line MCF7, the major lipogenic regulatory factor, sterol regulatory element binding protein 1c (SREBP1c), is upregulated by progesterone, which in turn stimulates the expression of the lipogenic genes acetyl CoA carboxylase (*ACC*) and fatty acid synthase (*FAS*) (20). In addition, studies have demonstrated a regulatory effect of progesterone on another lipogenic enzyme, lipoprotein lipase (LPL) in the mammary gland (21), which in turn may change the availability of long-chain fatty acids to the mammary gland cells.

In dairy cows, plasma progesterone concentration varies along the 21-day estrous cycle, which has two main phases. The follicular phase is characterized by a large diameter preovulatory follicle (>15 mm), high estradiol concentration, and low progesterone concentrations (0.5–1 ng/ml) in the plasma. The luteal phase starts after ovulation and is characterized by increased plasma progesterone concentration through the first 7 days of the estrous cycle. On days 8–10 of the cycle, the progesterone concentration peaks (4–6 ng/ml) and remains at this level until days 16–17 of the cycle (22). Thereafter, the progesterone concentration declines to basal levels, to enable new ovulation and initiation of a new estrous cycle. In the case of successful fertilization, the progesterone level remains high and stable to support the developing embryo and pregnancy. It has been shown that plasma progesterone concentration through the estrous cycle does not affect total milk fat content (23). However, more in-depth analyses of the structure and composition of milk fat were not conducted.

In the current study, we used *in vivo* and *in vitro* models to explore the involvement of progesterone in the regulation of MFG size. In particular, we examined whether changes in progesterone concentration through the estrous cycle are associated with MFG size.

## Materials and Methods

### Study Design

The study had two main parts: (1) *in vivo* study to examine the association between plasma progesterone concentration and the MFG size in milk. This examination was performed through an entire synchronized estrous cycle; (2) *in vitro* model to study the differential effect of progesterone concentration, in particular the changes in MFG size and composition upon exposure to increased progesterone concentration, mimicking the luteal phase, and decreased progesterone concentration, mimicking the follicular phase of the estrous cycle.

### Materials

MECs were cultured in basic DMEM/F12 medium (Biological Industries, Beit Haemek, Israel) supplemented with hyaluronidase, insulin, hydrocortisone, prolactin, bovine serum albumin, heparin, triolein, oleic acid, progesterone, Nile red, DAPI (Sigma Aldrich Israel, Rehovot, Israel), collagenase (Worthington Biochemical, Lakewood, NJ), trypsin, fetal bovine serum, streptomycin, amphotericin, glutamine (l-glutamate solution), chloroform, methanol, ethanol, and Trypan blue (Biological Industries). For the lipid analysis, we used analytical reagent-grade petroleum ether (Gadot Lab Supplies, Netanya, Israel), sulfuric acid (H_2_SO_4_; Diagnostic Products, Los Angeles, CA), chloroform, methanol, and ethanol analytical reagents (Bio-Lab, Jerusalem, Israel), and dichloromethane and methanol for liquid chromatography (Merk KGaA, Darmstadt, Germany). For the *in vivo* experiment, we used GnRH (Gonadorelin), prostaglandin (PGF2α; Parnell Laboratories, Sydney, Australia), and the Progesterone RIA kit (Medison Pharma, Petach Tikva, Israel).

### *In vivo* Study

The study was conducted on Holstein dairy cows at the dairy farm of the Agricultural Research Organization's Volcani Center according to the Ethics Committee of the Hebrew University. The selected animals (*n* = 12) were non-pregnant lactating cows, cyclic and healthy, over 100 days in milk. Cows were synchronized according to the “OvSynch” protocol which includes 2 ml intramuscular injection of GnRH analog (day 0), followed by injection of 2.5 ml PGF2α on day 7 and a second injection of 2 ml GnRH 48 h later. The second GnRH injection was defined as day −1 of the synchronized cycle. An additional PGF2α injection was carried out at the end synchronization to confirm ovulation. Follicle and corpus luteum development through the synchronized cycle were recorded by ultrasound monitoring (Aloka, SSD-900, Tokyo, Japan).

Blood and milk samples were collected daily from day 3 pre-estrus to day 2 post-estrus, and from day 17 to day 21 post-estrus. Blood samples were collected from the coccygeal blood vessel into vacuum tubes (Becton Dickinson Systems, Crowley, UK). The plasma was immediately separated by centrifugation for 10 min at 800× g and stored until analysis at −20°C. Plasma was taken to determine progesterone concentration using the Progesterone RIA kit according the manufacturer's protocol. Cows were milked three times a day and the lactation data, including milk yield, fat, protein, and lactose concentrations, were recorded automatically by the Afilab system (Afikim, Israel). Milk samples from the morning milking were stained with Nile red and MFG size was determined (detailed below).

### *In vitro* Study

Primary culture of MEC was performed according to a protocol established in our laboratory (12). Briefly, mammary tissue was collected from lactating cows in a commercial slaughterhouse and immediately transferred to ice-cold growth medium with 1,000 U/ml penicillin, 1 mg/ml streptomycin, 2.5 μg/ml amphotericin mixture, and 0.02 mg/ml heparin. Mammary tissue was digested in medium with 1 mg/ml collagenase, 1 mg/ml hyaluronidase, and 0.02 mg/ml heparin, for 3 h at 37°C. After incubation, the suspension was filtered through a metal mesh (250 μm). Sediments were treated with trypsin–EDTA solution and filtered through a 100-μm cell strainer (BD Falcon, Bedford, MA). Then, the cells were grown in plastic culture dishes with DMEM/F12 supplemented with 10% (*w*/*v*) fetal bovine serum, 100 U/ml penicillin, 100 μg/ml streptomycin, 0.25 μg/ml amphotericin B, 1 μg/ml insulin, and 0.5 μg/ml hydrocortisone. The medium was changed every 48 h.

### Experimental Design

To examine the effect of progesterone on MFG formation, secretion, and size, the culture medium was supplemented with very-low-density lipoprotein (VLDL) and progesterone at different concentrations (0, 1, 5, and 20 ng/ml). Progesterone concentrations were based on those found in the plasma during the luteal and follicular phases *in vivo*. After 24 h, cells were either fixed for lipid droplet size analysis (detailed below (or harvested with trypsin (0.05% *w*/*v*), sedimented by centrifugation, and washed with 0.9 g/L NaCl.

To determine whether the absolute progesterone concentration or its pattern (i.e., increasing or decreasing) affects MFG, cells were treated for 48 h with increasing doses of progesterone: 0.75, 2.5, and 5 ng/ml. The culture medium was replaced after 11, 17, and 20 h, respectively, aimed to mimic the *in vivo* luteal phase. The decreasing phase consisted of exposure to 5, 2.5, and 0.75 ng/ml progesterone. Culture medium was replaced after 8, 12, and 28 h, respectively, to mimic the follicular phase.

To determine whether the effect of progesterone on lipid droplet size is mediated by the presence of VLDL, a b-factorial experiment was performed. MECs were cultured with increasing or decreasing progesterone concentrations in the presence or absence of VLDL in the culture medium. This study was aimed to provide indirect evidence of LPL activity, acting on VLDL and providing the MEC with exogenous, preformed fatty acids. After 48 h of incubation, cells were fixed and stained with Nile red and intracellular lipid droplet size was determined.

### Lipid Extraction and Chromatographic Analysis

For lipid extraction and analysis, 150,000 MECs were plated in a 60-mm plastic dish. For Nile red fluorescence staining, 50,000 MECs were plated in a six-well plate on glass cover slips. After 24 h of incubation, the culture medium was replaced with DMEM/F12 without serum, containing 0.15% (*w*/*v*) free fatty acids–free BSA and insulin (1 μg/ml), hydrocortisone (0.5 μg/ml), and prolactin (1 μg/ml). Cells were incubated for 48 h to induce milk lipid and protein synthesis.

Lipids were extracted by the Folch protocol (24). Briefly, each cell sample was incubated for 1 h with “Folch mixture” (chloroform/methanol, 1:2 volumetric ratio). The organic phase was separated by addition of double-distilled water (DDW) and overnight incubation at 4°C. The upper phase was then removed and the lower phase filtered through glass wool. The lower phase was evaporated under nitrogen and then dissolved in chloroform/methanol (3:97). Samples were kept at −20°C until further analysis.

Lipid separation was performed by thin-layer chromatography and gas chromatography analysis. For the thin-layer chromatography, silica gel was spread on glass plates and activated at 105°C, 24 h before the analysis. After cooling, the cell samples, extracted by Folch protocol, were loaded into the plates along with Tg and PL standards. The plates were inserted into a tank with a mixture of petroleum ether, water, and acetic acid (8:2:1 volometric ratio) for 30 min, then sprayed with 0.05% *v*/*v* dichlorofluorescein in ethanol) and examined under UV light. The detected lipid segments were then separated from the plates into methylation mixture (5% *v*/*v* H_2_SO_4_ in methanol) for 1-h incubation at 65°C. Then, 1.5 ml petroleum-ether and 3 ml DDW were added to each sample. The upper phase was collected into a new vial and the organic solvent was evaporated with nitrogen. Then 100 μl petroleum-ether was added to each sample for fatty acid analysis in a gas chromatograph model 6890N, equipped with a flame-ionization detector and DB-23 capillary silica (0.25-μm film; Agilent Technology, Wilmington, DE). The analysis was performed according to a protocol established in our laboratory (25). Peaks were identified with Chemstation software (Agilent Technology). The concentrations of fatty acids are given as molar percentages of the total molar sum of the identified fatty acids. Fatty acids were classified according to chemical characteristics such as double bonds and carbon-chain length.

High-pressure liquid chromatography (HPLC) was performed in an HPLC 1200 (Agilent Technology) equipped with evaporative light-scattering detector. Tg, cholesterol, and PL were identified using external standards (Sigma Aldrich). Quantification was performed against external standard curves and expressed as micrograms per 10^6^ live cells or as weight percent of the total PL (μg) in the sample. The number of live cells was determined with a hemocytometer after Trypan blue staining.

### Fluorescence Staining of Lipid Droplets and MFG

#### Mammary Epithelial Cells

Cells were grown on glass cover slips, washed three times with phosphate-buffered saline (PBS) and fixed with 4% paraformaldehyde in PBS for 20 min at room temperature. Then the cells were washed four times with PBS, stained with Nile red (200 nM), and incubated for 15 min. Cover slips were then washed three times with PBS and stained with DAPI for 5 min. Cover slips were washed four more times with PBS and mounted on a slide with fluorescent mounting medium (Dako North America, Carpinteria, CA). The slides were visualized under an Olympus BX40 fluorescence microscope equipped with an Olympus DP73 digital camera using CellSens Entry software (version 1.7; Olympus). Lipid droplet diameter was measured using ImageJ software (version 1.48; NIH, Bethesda, MD). Lipid droplet diameter was divided into three size categories: small (<1 μm), medium (1 μm < *x* < 2 μm), and large (>2 μm).

#### Milk Staining

Milk samples were stained with Nile red in acetone (42 μg/ml) for 2 h at room temperature. For fixation, agarose was dissolved in DDW (5 mg/ml) and mixed with the milk sample and dye at a 1:20 ratio. The samples were visualized under a fluorescence microscope. Lipid droplet diameter was measured and MFG were characterized individually for each cow and day of estrus. The MFG were divided into two size categories: small (<3 μm) and large (>3 μm).

### Statistical Analysis

The statistical procedures were performed using JMP software version 12.0.1 (SAS Institute, Cary, NC). Experimental results were analyzed by one-way ANOVA. All dependent variables were checked for homogeneity variance by unequal variances in JMP software and if the variance was not homogeneic, a Welch ANOVA test was performed. Comparisons were made by ANOVA followed by Tukey–Kramer HSD test. The distribution of cell phenotypes based on lipid droplet size categories was compared by χ^2^-test followed by Fisher's exact test. Significant probability was set to 0.05 and tendencies were reported at 0.05 < *p* ≤ 0.1.

For plasma progesterone concentration, milk yield, and concentration of fat, protein, and lactose in milk, a repeated measures ANOVA was used. Days post-estrus was defined as the covariate. For differences in milk solids and MFG size between increasing and decreasing phases of progesterone concentration during the estrous cycle, the results of progesterone concentration throughout the estrous cycle were plotted for each cow individually. The increasing phase was determined as the period during the estrous cycle in which elevated progesterone concentration was observed, whereas the rest of the estrous cycle was considered the decreasing phase. The distribution of MFG size categories was compared by χ^2^-test followed by Fisher's exact test. All data are reported as means ± SEM. Significance was set at *p* < 0.05.

## Results

### *In vivo* Study

#### Milk Yield and Contents

**Figure 1:**
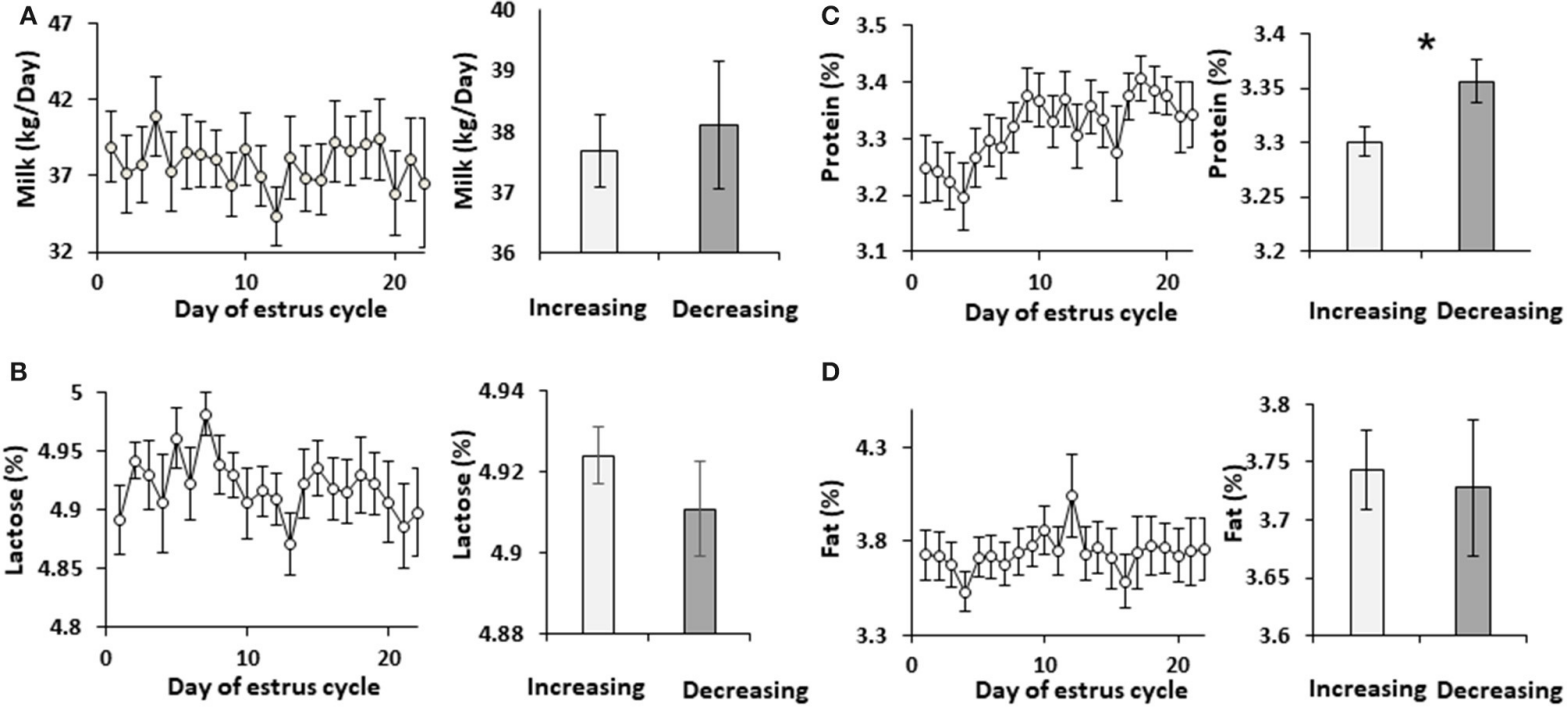
Milk yield and solids concentration through the estrous cycle. The daily values of milk yield and solids concentrations, and the mean values during the increasing and decreasing progesterone concentration phase throughout the estrous cycle are presented: **(A)** milk yield (L/day); **(B)** concentration in milk of lactose (%); **(C)** concentration of protein (%); **(D)** concentration of fat (%). No differences were found throughout the estrous cycle in milk yield (*p* = 0.97) or in the concentration of lactose, protein, and fat (*p* =0.79, 0.21, and 0.98, respectively). No differences were observed when the mean values of milk yield and the concentrations of lactose and fat between the increasing and decreasing phase of progesterone concentrations were compared (*p* = 0.71, 0.3, 0.8, respectively). However, protein concentration was lower in the increasing phase of progesterone concentration of the estrous cycle compared with the decreasing phase (*p* =0.019). *indicates significant difference, *P* < 0.05.

Cows were administered synchronized intramuscular injection of GnRH analog. Milk was sampled daily throughout the estrous cycle and analyzed. Neither milk yield nor lactose concentration changed during the estrous cycle. Accordingly, these parameters did not differ between the increasing and decreasing phases of progesterone concentration (Figure 1). Milk protein concentration did not change during the estrous cycle, but it was 10% higher in the decreasing vs. increasing phase of progesterone concentration (*p* = 0.02; Figure 1).

#### Corpus Luteum Development and Plasma Progesterone Concentration

**Figure 2:**
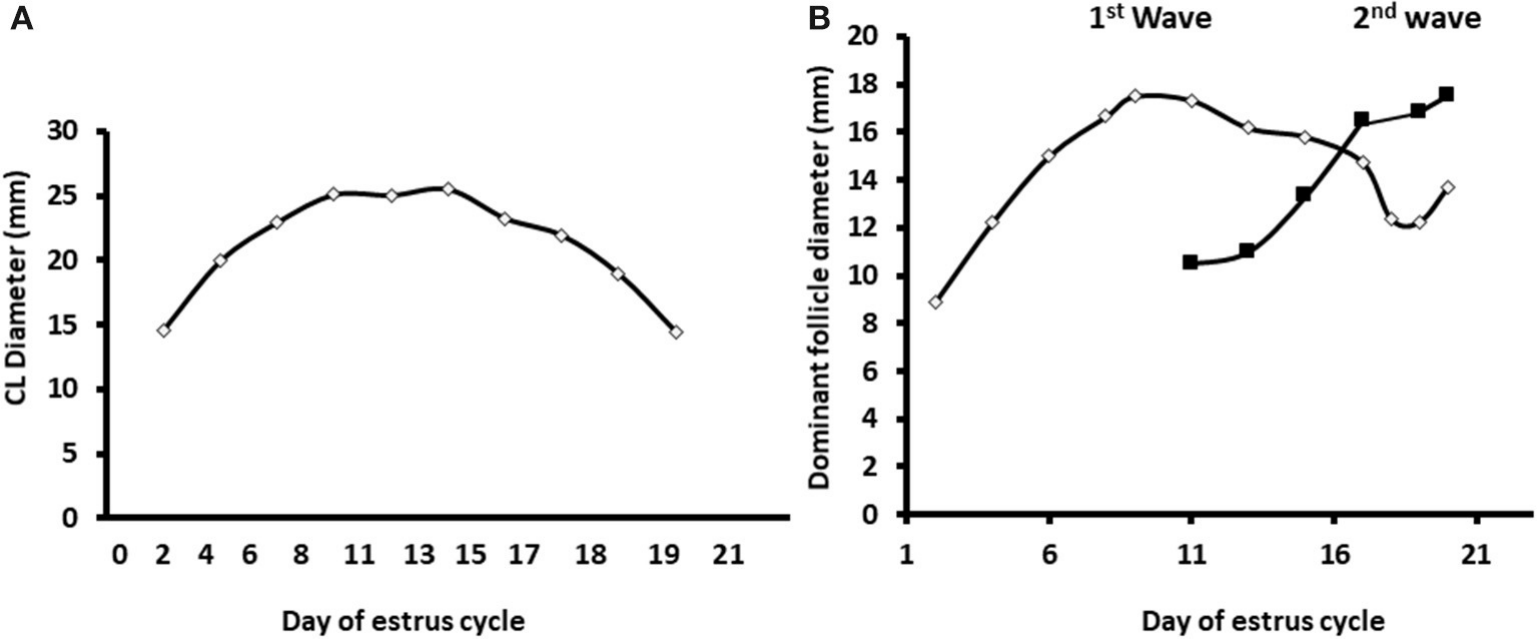
Diameter of the developing corpus luteum and the dominant follicles through the estrous cycle. The data displayed as the average diameters measured in dairy cows along the estrous cycle (*n* = 12) of the corpus luteum and largest follicle in the two follicular surges during a normal estrous cycle. **(A)** Daily average diameter of the corpus luteum (CL) along the estrous cycle. **(B)** Average diameter of the dominant follicles during two subsequent follicular surges during the estrous cycle. Mean diameter of the largest follicle during the first and second follicular surge—white and black, respectively. Values represent mean ± SE.

Ultrasonographic scanning indicated a normal pattern of corpus luteum formation and regression. The follicular developmental pattern was normal as well, expressed by two follicular waves and development of dominant and preovulatory follicles in the first and second wave, respectively (Figure 2).

**Figure 3:**
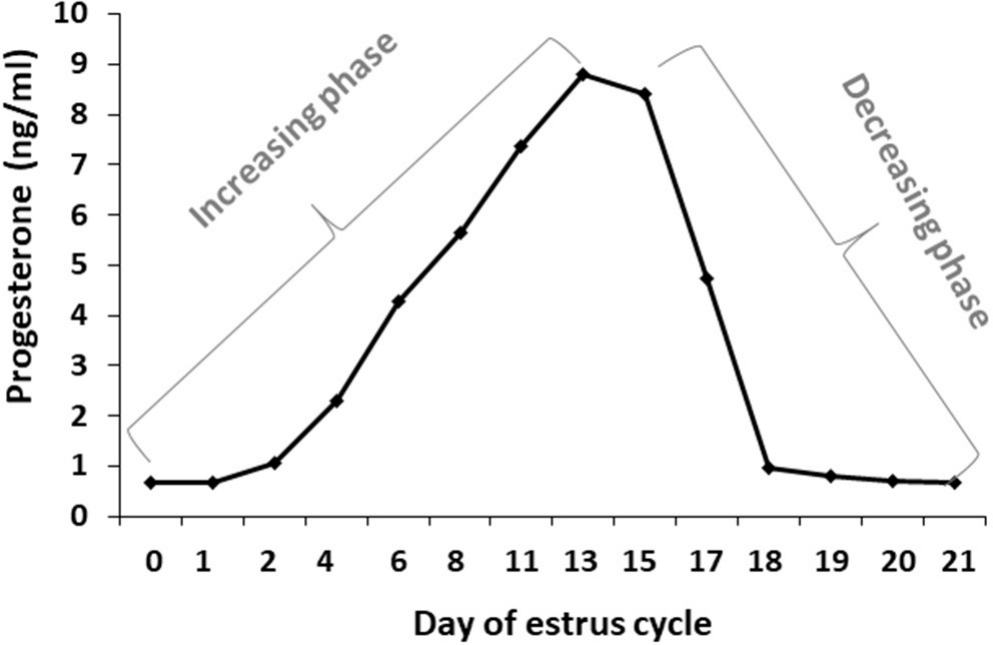
Daily progesterone concentrations throughout the estrous cycle. Average progesterone concentrations (ng/ml) in daily blood samples of Holstein cows (*n* = 12) was determined during the estrous cycle. The first part of the estrous cycle shows increasing progesterone concentrations (days 0–13). A decrease in the progesterone levels was observed from day 16 post-ovulation.

Blood samples were used to determine progesterone concentrations throughout the estrous cycle using RIA. In addition, the progesterone concentrations were used to determine for each cow, individually, the increasing and decreasing progesterone phase. Progesterone concentrations from days 0 to 13 of the estrous cycle increased from 0.67 to 8.8 ng/ml, respectively, defined as the increasing phase. Progesterone concentrations from day 15 to days 20–21 decreased to basal level, defined as the decreasing phase (Figure 3).

#### Daily MFG Number and Diameter During the Estrous Cycle

**Figure 4:**
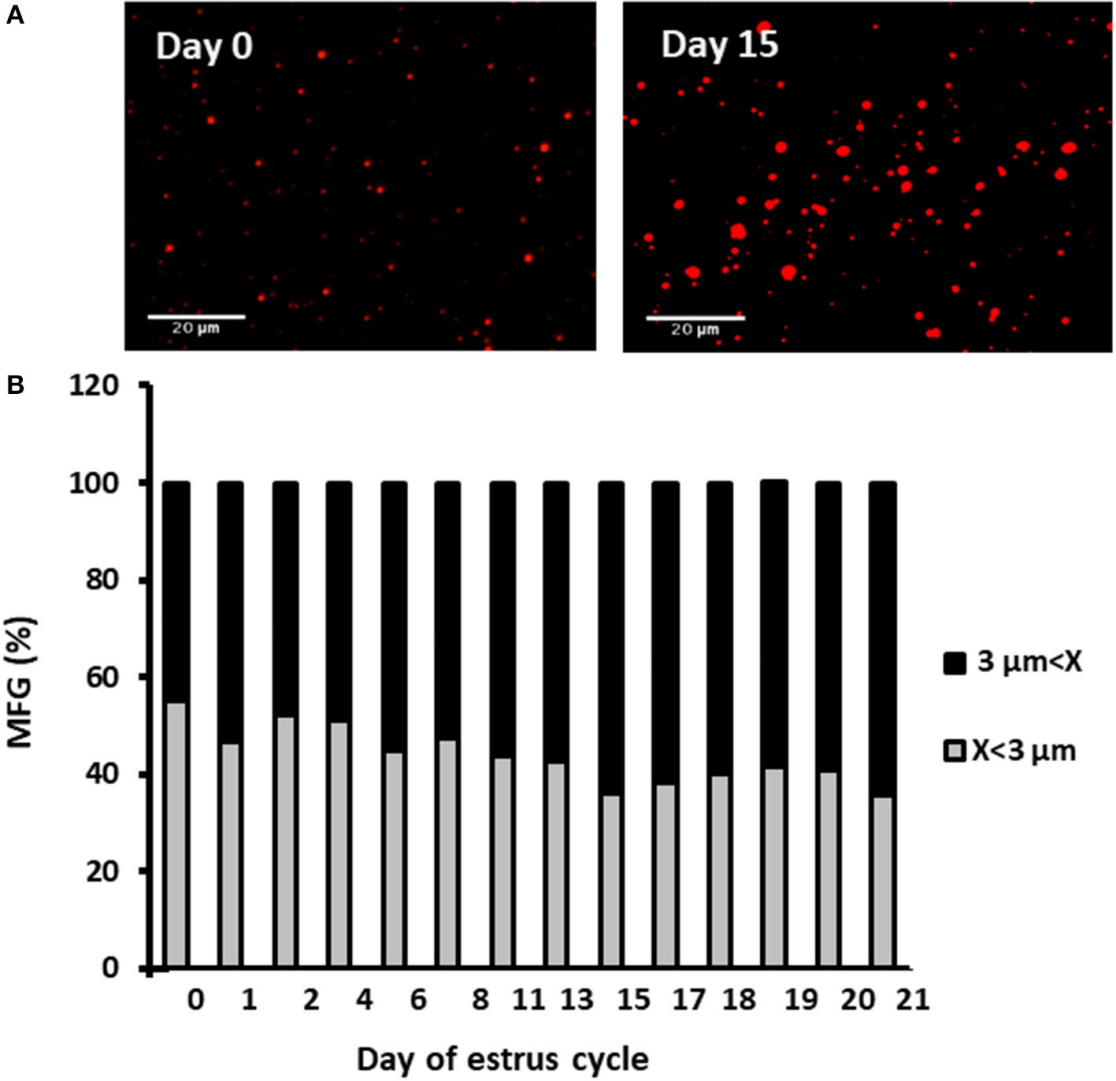
Diameter and size distribution of milk fat globules (MFGs) throughout the estrous cycle. Milk samples were collected daily throughout the estrous cycle and fat globules stained with Nile red and measured. **(A)** Representative images taken with fluorescence microscope of whole milk, demonstrating the size differences of MFGs (red) between the day of ovulation (day 0) and 15 days post-ovulation. **(B)** Distribution of MFG size along the estrous cycle. Representative fields (*n* = 3–5) were analyzed for each milk sample collected (*n* = 12) at each day of the cycle. The measured MFGs were divided into size categories: small lipid droplets (*x* < 3 μm; gray column) and large lipid droplets (*x* > 3 μm; black column). Differences between size distribution throughout the cycle were measured by χ^2^-test (*p* ≤ 0.05).

**Figure 5:**
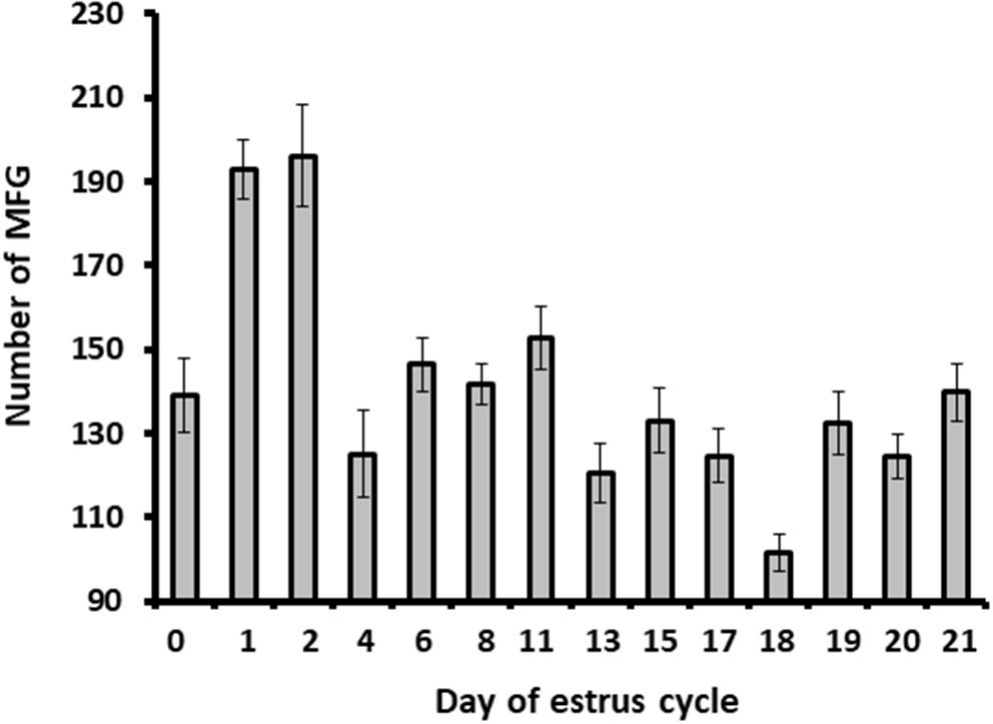
The MFG number throughout the estrous cycle. Milk samples were stained with Nile red and the milk fat globule (MFG) number was recorded. Five microscopic fields were counted for each sample (i.e., from each cow; *n* = 12). The MFG number varied between 101 and 196 droplets per sample along the estrous cycle. Higher number of MFG was observed on days 1 and 2 post-ovulation (*p* ≤ 0.0001).

Milk samples were stained with Nile red to determine their diameter and number. The average MFG diameter changed during the estrous cycle (21 days) from 2.9 to 3.6 μm. The distribution of MFG into large and small globules, based on their diameter, indicated a dominant phenotype of small MFG (<3 μm) through the increasing progesterone phase. On the other hand, the decreasing progesterone phase was associated with a high proportion of large MFG (>3 μm; *p* < 0.0001, Figure 4). Along the estrous cycle, the number of MFG varied between 101 and 196 MFG per sample. It should be noted that days 1 and 2 post-ovulation were characterized by a unique and significant increase in the number of MFG (Figure 5).

**Figure 6:**
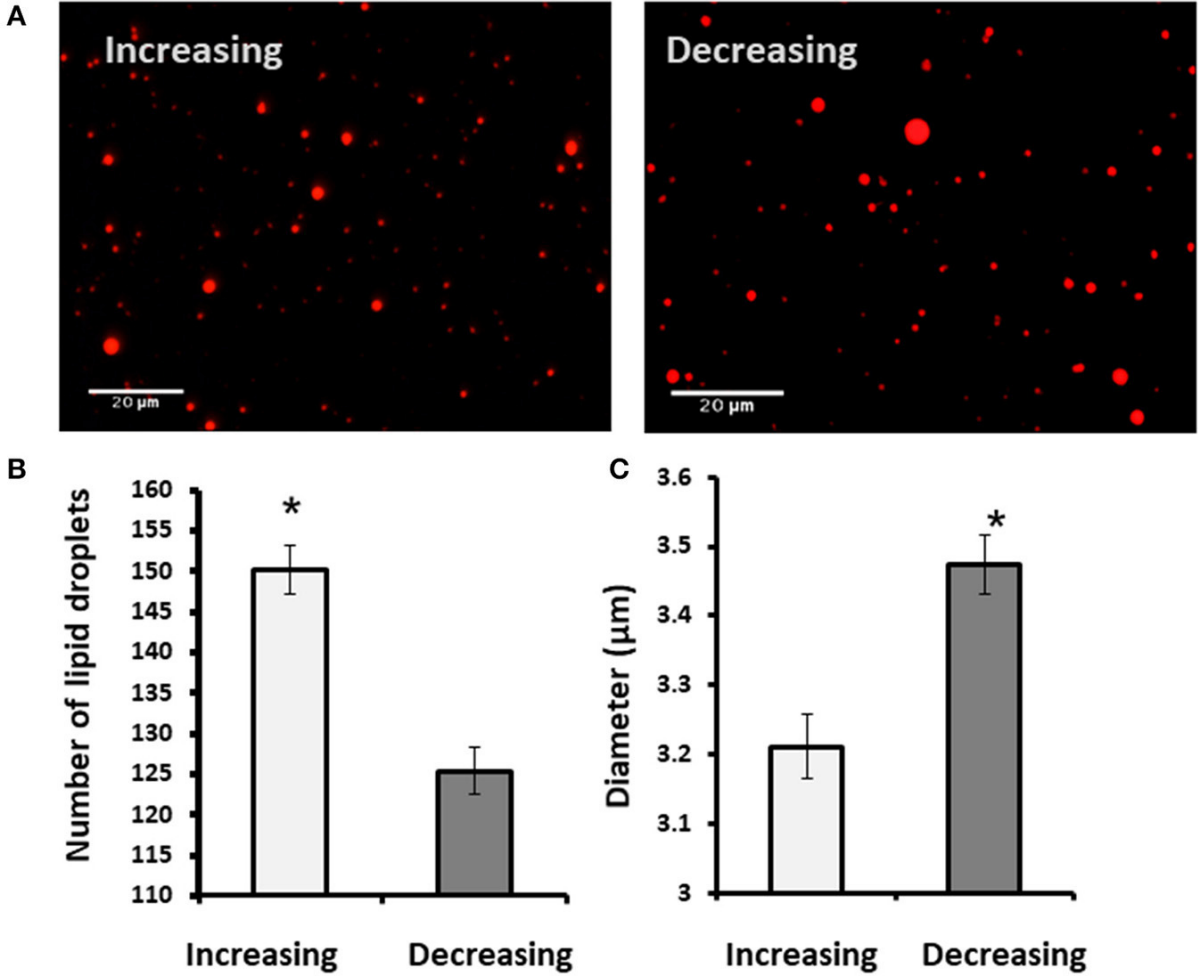
The MFG morphometric traits during the increasing and decreasing phase of progesterone concentration. Milk samples were stained with Nile red which incorporates into the MFG and enables their visualization under fluorescence microscope to determine size and number of MFG. **(A)** Representative images of the MFG during the increasing or decreasing progesterone concentration phase through the estrous cycle. **(B)** Number of MFG droplets during the increasing (white) and decreasing (gray) progesterone concentrations. During the increasing phase, MFG number was elevated by 20% comparing the decreasing phase. **(C)** The average diameter of the MFG during the increasing (white) and decreasing (gray) of the progesterone concentrations. The average diameter during the increasing phase was lower by 9% relative to that in the decreasing phase. Asterisk represents significant differences, *p* ≤ 0.0001.

When the estrous cycle was divided into increasing and decreasing progesterone concentration phases (Figure 6), it was found that during the increasing phase, MFG size decreased by 9% compared with that during the decreasing phase.

### *In vitro* Study

#### Number of Lipid Droplets

**Figure 7:**
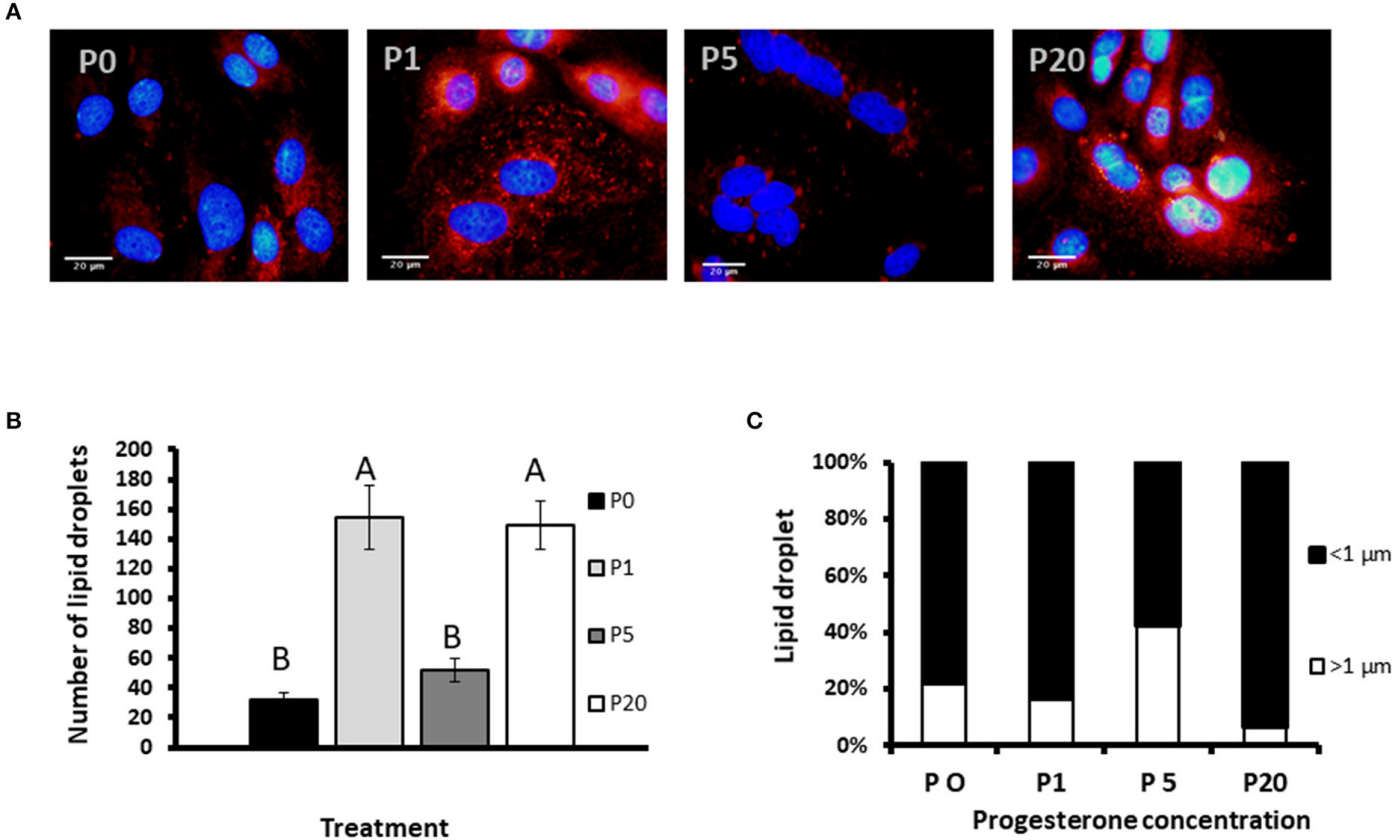
The effect of progesterone on morphometric traits of intracellular lipid droplets in mammary epithelial cells (MECs). MECs were treated with progesterone at 0, 1, 5, and 20 ng/ml (P0, P1, P5, and P20, respectively) for 24 h. After incubation, nucleus was stained with DAPI (blue) and lipid droplets with Nile red (red). Cells were visualized under fluorescence microscope. The number and size of lipid droplets were recorded. **(A)** Representative pictures of MECs and lipid droplets treated with different progesterone concentrations. Scale bars, 20 μm. **(B)** Number of lipid droplets per treatment. Data presented as mean ± SEM and different letters indicate differences between treatment groups at significant levels of *p* ≤ 0.0001. **(C)** Distribution of lipid droplet size. Lipid droplets were divided to size categories; small: *x* < 1 μm (black), large: *x* > 1 μm (white). Nine representative fields (10 to 30 cells per field) were analyzed for each replicate (*n* = 3 replicates per treatment). Differences between treatments were determined by χ^2^-test (*p* ≤ 0.05).

We used primary culture of MEC to determine progesterone effect on the size of the lipid droplets. Lipid droplets were visualized and size determined before their secretion from MEC, and we used their morphometric traits as a proxy for the secreted MFG. To determine the effect of progesterone on lipid droplets, intracellular LD were stained with Nile red and the nucleus was stained with DAPI (Figure 7). The number of lipid droplets found in cells treated with 1 or 20 ng/ml progesterone was 3.5-fold higher than in the control (no progesterone; *p* < 0.0001). The number of lipid droplets in cells treated with 5 ng/ml progesterone did not differ from the control.

#### Effect of Progesterone on Lipid Droplet Size

To examine the effect of progesterone on lipid droplet size, MECs were fixed and stained with fluorescent dye. Lipid droplets were divided into two main categories based on their size: large (>1 μm) and small (<1 μm), as previously described (12) (Figure 7C). The distribution of lipid droplets in the size categories was affected by progesterone (*p* < 0.0001). At 5 ng/ml progesterone, over 40% of the droplets were larger than 1 μm, whereas at 0, 1, or 20 ng/ml progesterone, the proportion of large droplets did not exceed 20%. In particular, culturing cells with 20 ng/ml progesterone resulted in the lowest rate of large droplets, only 6.5%.

#### Effect of Progesterone on Membrane Lipid Composition and Concentration

**Figure 8:**
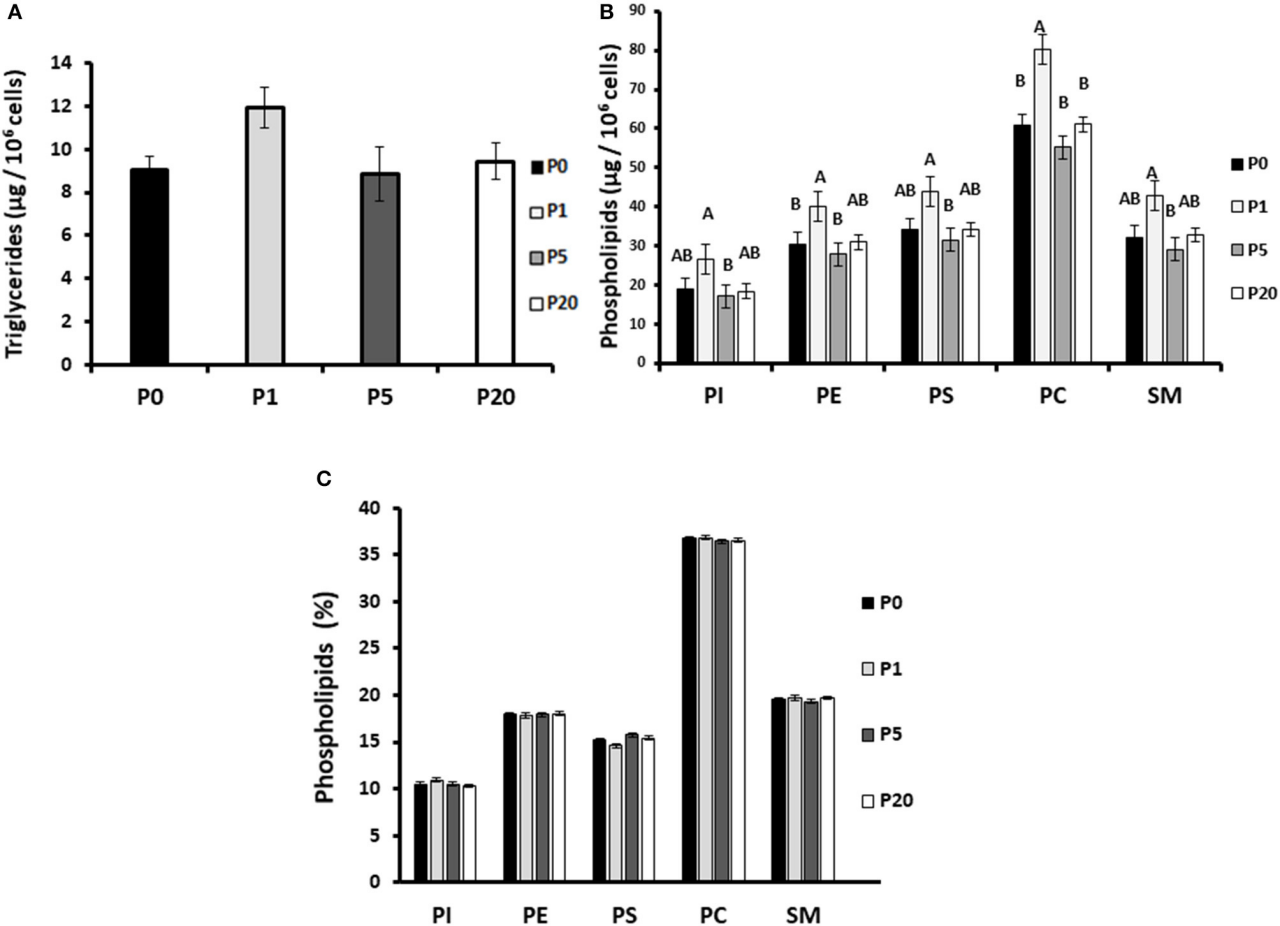
Progesterone affects lipid content and composition in mammary epithelial cells. Mammary epithelial cells were treated with progesterone at 0, 1, 5, and 20 ng/ml (P0, P1, P5, and P20, respectively) for 24 h. After treatment, cells were harvested and PL content and composition was determined by HPLC-ELSD. **(A)** Cellular triglyceride content did not differ between treatments. **(B)** Phospholipid concentration in mammary epithelial cells was determined. Cells treated with 1 ng/ml progesterone had greater PL content whereas cells treated with 5 ng/ml progesterone had lower phospholipid content relative to the control. **(C)** Phospholipid composition (weight %) in the mammary epithelial cells did not differ between treatments. PI, phosphatidylinositol; PE, phosphatidylethanolamine; PS, phosphatidylserine; PC, phosphatidylcholine; SM, sphingomyelin. Data are presented as mean ± SD. Different letters indicate significant differences in the level of *p* ≤ 0.05.

To reveal the potential mechanism by which progesterone affects the number and diameter of lipid droplets, cellular lipids were extracted and their composition determined. In particular, the composition of the five major membrane PL (PI, PE, PS, PC, and SM) was examined (Figure 8). Progesterone concentration did not affect the membrane PL composition.

The concentration of Tg was not affected by progesterone (Figure 8A). It should be noted, however, that incubation of MEC with 1 ng/ml progesterone increased the concentration of the measured PL by approximately 1.3-fold, compared with 0, 5, and 20 ng/ml progesterone (*p* < 0.05). The greatest effect of progesterone was observed on PC, expressed as an elevated concentration−20 μg/10^6^ cells (*p* < 0.05). On the other hand, incubation of MEC with 5 ng/ml progesterone tended to decrease PL amount (*p* < 0.05).

#### Effect of Progesterone on MEC Fatty Acid Profile

**Figure 9:**
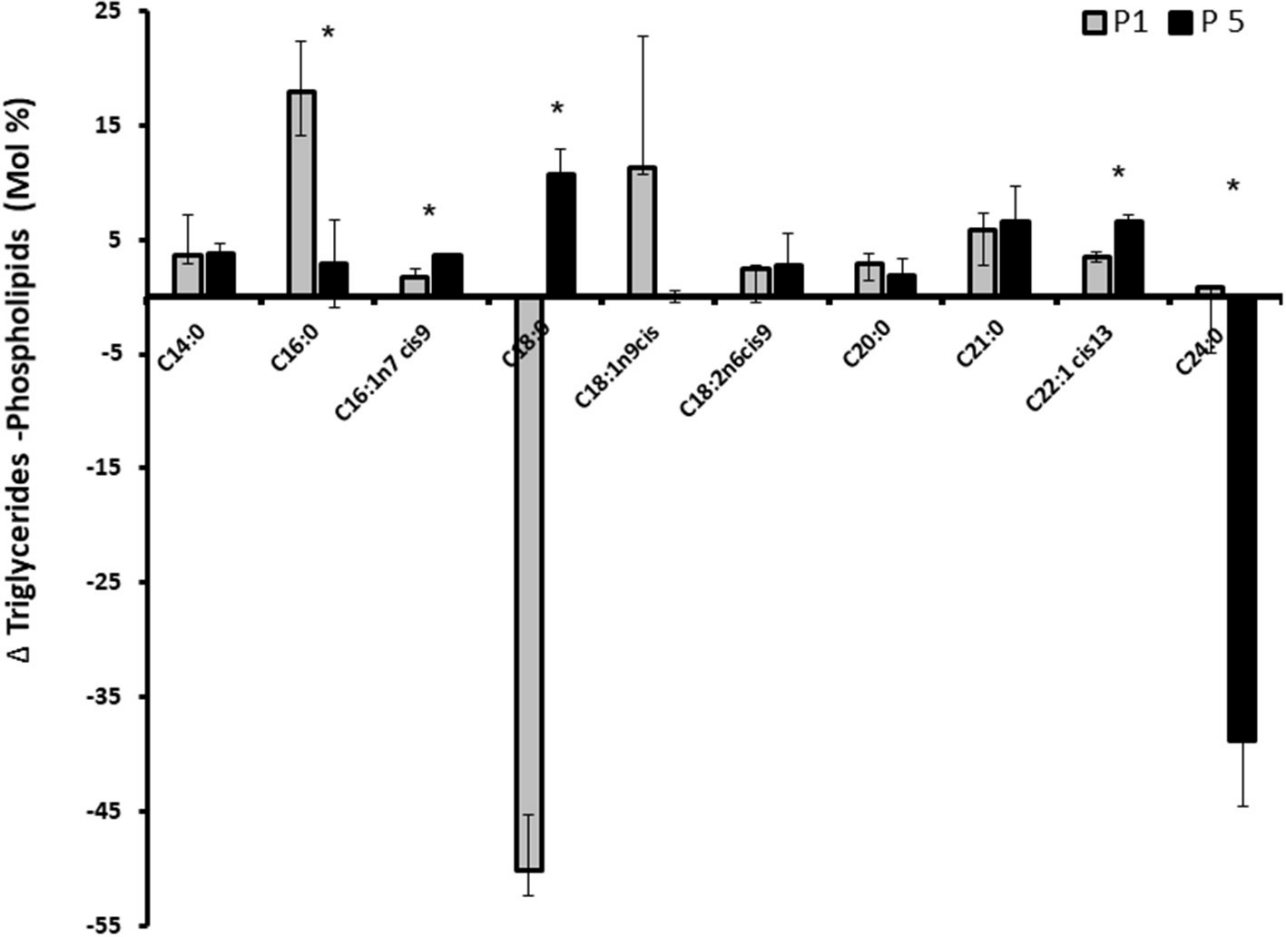
Effect of progesterone on mammary epithelial cells fatty acids profile. Mammary epithelial cells were treated with progesterone at 1 and 5 ng/ml (P1 and P5, respectively) for 24 h. After treatment, cells were harvested and their fatty acid composition was determined. The concentration of each fatty acids is presented as the delta between its concentration in triglyceride and its concentration in the phospholipid fraction. Positive values indicate greater utilization for fat synthesis (triglyceride) whereas negative values indicate greater utilization for membrane (phospholipid) synthesis. Data are presented as mean ± SD. Asterisk indicates significant differences in the level of *p* ≤ 0.05.

We assessed whether progesterone concentration pattern is involved in fatty acid utilization for membrane or Tg synthesis. After incubating the cells with 1 or 5 ng/ml progesterone, total fat was extracted and separated to neutral (Tg) and polar lipids. Fatty acid composition in each lipid fraction was determined. The relative fatty acid concentration is demonstrated as the delta between Tg and PL (Figure 9). The concentration of stearic acid (C18:0) was higher in the PL compartment in cells treated with 1 ng/ml progesterone. The concentration of lignoceric acid (C24:0) was higher in the PL compartment in cells treated with 5 ng/ml progesterone (*p* < 0.05).

#### Effect of Progesterone Concentration Pattern on Lipid Droplet Number, Diameter, and Size Distribution

**Figure 10:**
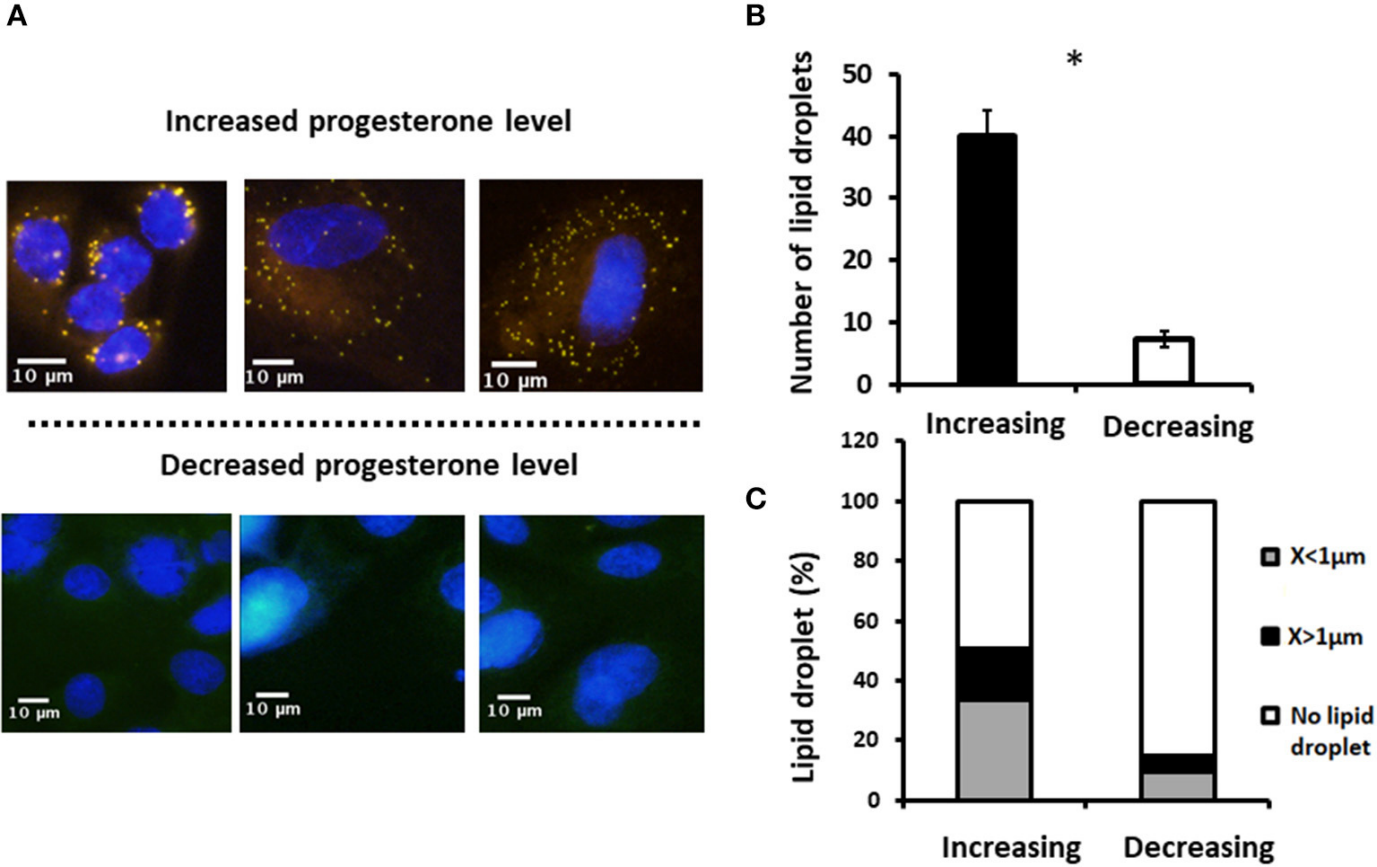
Incubation of mammary epithelial cells with increasing or decreasing progesterone concentrations changed the intracellular lipid droplet number and size. Mammary epithelial cells incubated with progesterone either in increasing (0.75, 2.5, and 5 ng/ml) or decreasing (5, 2.5, and 0.75 ng/ml) order. After incubation, nucleus was stained with DAPI (blue) and lipid droplets were stained with in Nile red (red) and their number and size recorded. **(A)** Representative figures from increasing and decreasing progesterone concentration treatments. Although at the end of the increasing treatment lipid droplets are easily visualized in the cytoplasm of the cells, no lipid droplets can be visualized after the decreasing treatment. **(B)** Number of lipid droplets after treatments with increasing or decreasing progesterone concentrations. Greater number of lipid droplets was counted in cells treated with increasing progesterone concentrations. **(C)** After treatment with increasing or decreasing progesterone concentrations, three phenotypes were examined: cells with large lipid droplets (*x* > 1 μm, black), cells with small lipid droplets (*x* < 1 μm, gray), and cells without lipid droplets (white). Cellular phenotype was designated according to the maximal diameter of its visualized lipid droplets. The large lipid droplet phenotype was only visualized in cells treated with increasing progesterone levels. Asterisk represents a significant difference between treatments at the level of *p* ≤ 0.0001.

To examine whether the effect of progesterone is associated with its concentration pattern, MECs were incubated with increasing (0.75, 2.5, and 5 ng/ml) or decreasing (5, 2.5, and 0.75 ng/ml) levels of progesterone—an *in vitro* model mimicking the progesterone curve through the estrous cycle *in vivo*. Culturing with increasing progesterone concentrations resulted in a 5.7-fold elevation in the number of lipid droplets relative to MECs treated with decreasing progesterone concentrations (*p* < 0.0001). In addition, the distribution of the lipid droplet sizes differed between the two patterns. In particular, increasing progesterone concentration was associated with a 3-fold increase in the large lipid droplets compared with the increasing progesterone pattern (*p* < 0.0001; Figure 10).

#### Effect of Progesterone on Lipid Droplet Size Requires the Presence of VLDL

**Figure 11:**
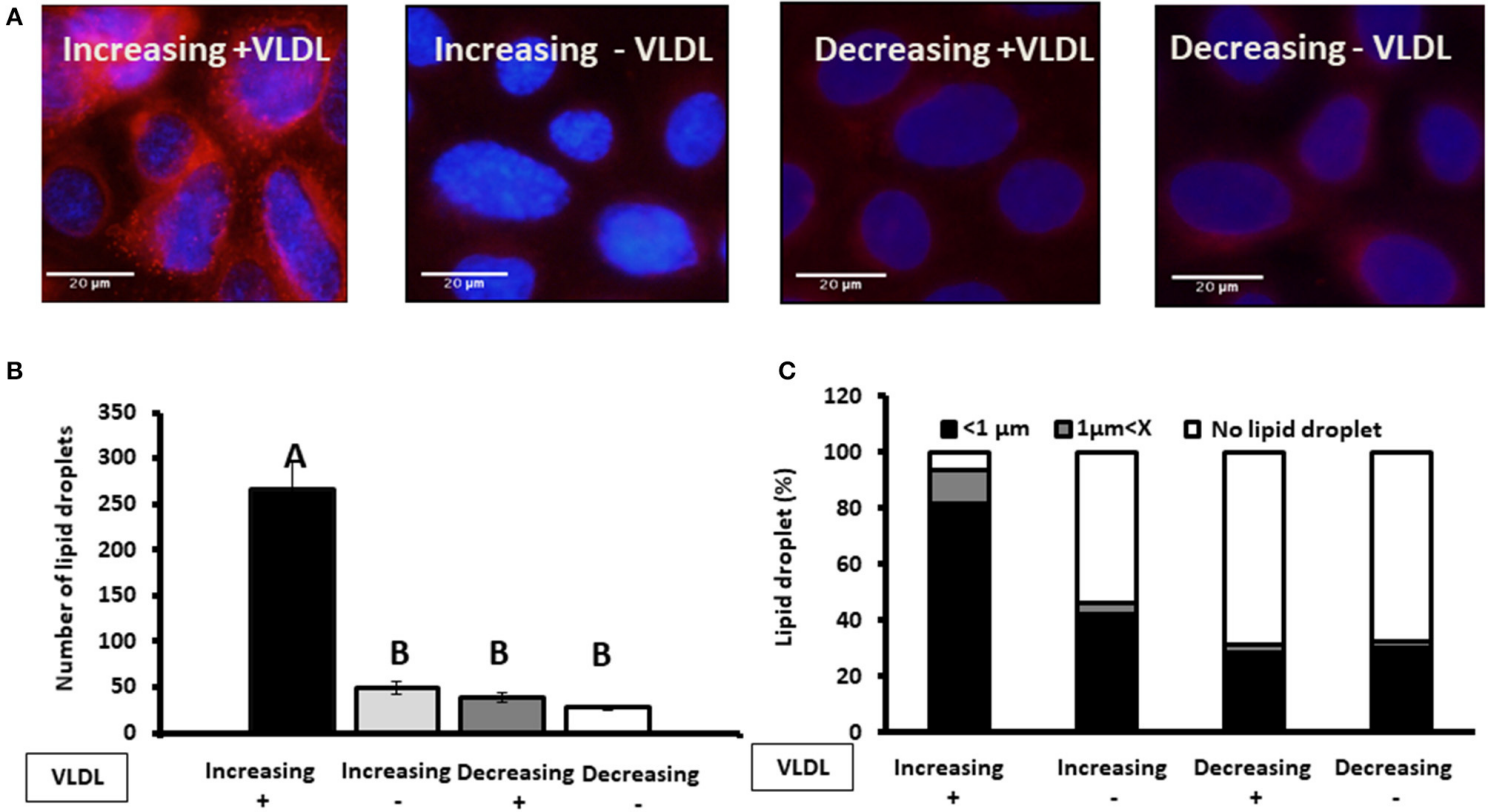
The effect of progesterone on the lipid droplet number is VLDL dependent. Mammary epithelial cells (MECs) were incubated with increasing (0.75, 2.5, and 5 ng/ml) or decreasing (5, 2.5, 0.75 ng/ml) progesterone concentration, with the presence or absence of VLDL. After incubation, nucleus was stained with DAPI (blue) and lipid droplets in Nile red (red). Lipid droplets were counted and measured. **(A)** Representative figures from increasing and decreasing progesterone concentration treatments with and without VLDL. The most pronounced lipid droplets were found in the increasing progesterone treatment, in the presence of VLDL. Differences between increasing and decreasing treatments were only presented if VLDL was added to the culture medium. **(B)** Average number of lipid droplets by treatment per field; *n* = 5 fields with 24–50 cells per field and 3 replicates per treatment. Highest number of lipid droplets was recorded when MECs were treated with increasing progesterone concentration and with VLDL. Different letters indicate differences between treatments (*p* ≤ 0.0001). **(C)** Cells were divided into three phenotypes according to the largest lipid droplet measured: cells with large lipid droplets (*x* > 1 μm, gray), cells with small lipid droplets (*x* < 1 μm, black), and cells without lipid droplets (white). The difference between treatment in the distribution of cells among 3 phenotypes was tested with χ^2^-test. When VLDL was excluded from the culture medium, no differences between cells incubated with increasing or decreasing progesterone levels were found (*p* ± 0.0001).

To examine whether findings related to the pattern of progesterone concentration are associated with VLDL, MECs were incubated with increasing or decreasing progesterone levels, in the presence or absence of VLDL in the medium (Figure 11). The differences in lipid droplet diameter and number were found to be VLDL dependent. In particular, using the increasing progesterone concentration model with VLDL, the proportion of MEC with small lipid droplets increased 2.4-fold. In addition, a 4-fold increase in MEC with large lipid droplets was recorded when cells were incubated with VLDL in the decreasing progesterone concentration model (*p* < 0.0001). On the other hand, in the absence of VLDL, no differences were found between cells incubated with increasing or decreasing progesterone levels. This was true for lipid droplet number and cellular phenotype (Figure 11; *p* < 0.0001).

## Discussion

Progesterone plays a pivotal role during mammogenesis (1) and serves as an inhibitor of the secretory activation of mammary cells in the last days of gestation (2). However, its role in lipid metabolism and the composition and content of milk fat during lactation has been less documented. In agreement with previous studies, the findings of the current study indicate that progesterone does not have any direct effect on milk production or fat content during the estrous cycle. On the other hand, our data provide new evidences, suggesting that progesterone is involved in the regulation of MFG size. Findings from both *in vivo* and *in vitro* models indicate that the concentration pattern and direction (increasing vs. decreasing), rather than the specific concentration, of plasma progesterone affects MFG size.

It is well-accepted that the reproductive hormones estradiol and progesterone do not affect milk production during the estrous cycle (23). In support of this, in the current study, neither milk production nor milk fat content changed throughout an entire estrous cycle. On the other hand, MFG size changed during the estrous cycle in association with plasma progesterone concentration. Moreover, a comparison of MFG sizes in the luteal and follicular phases revealed a clear association between size and the pattern of plasma progesterone concentration. The proportion of small lipid droplets (<3 μm) was higher in the luteal phase which is characterized by increasing progesterone concentrations, whereas in the follicular phase, characterized by decreasing progesterone concentration, a high proportion of large lipid droplets (>3 μm) was recorded in milk. Although not clear cut, a prominent increase in the proportion of small MFG was found on the day of estrus (day 0 of the cycle). This phenotype might be associated with the decrease in progesterone concentration on the previous days, rather than with a direct effect of the basal low progesterone concentration on the day of estrus itself. This assumption is supported by the findings of the *in vitro* part of our study.

MFG size is determined before its secretion from MEC, during the migration of its precursors, the intracellular lipid droplets, from the site of their synthesis to the site of their secretion, the apical pole of the cell. The initial synthesis and the size regulation of the lipid droplets are common to many of the lipogenic tissues, such as adipose, liver, and mammary gland (26). Several mechanisms have been suggested to control lipid droplet size in MEC, adipocytes, and hepatocytes, such as fusion of intracellular lipid droplets (12–14), and the lipogenic capacity of the cell vs. the extent of lipolysis. For example, in 3T3-L1 adipocytes, cAMP-induced lipolysis reduced lipid droplet size (15). Some proteins that might regulate lipolysis and hence lipid droplet size include the lipid droplet-binding protein CGI-58, which activates adipose Tg lipase and hence reduces droplet size (27), and the G0/G1 switch protein (G0S2) and perilipin 5, which inhibit this same lipase and hence increase droplet size (28, 29). Adipophilin may also play a role in size regulation, as mice deficient in adipophilin failed to produce large lipid droplets in their mammary gland (30). Perilipin A, located on the lipid droplet surface, also restricts the access of lipases, thus increasing lipid droplet size (31). Under fasting conditions, perilipin A is phosphorylated and removed from the lipid droplet, allowing lipases to adhere to the lipid droplet surface and stimulate Tg hydrolysis (32). Taken together, lipolysis and lipogenesis are mostly regulated by metabolic signals, and largely associated with the total fat content in the cell. Nevertheless, the results of the present study did not show any direct effect of progesterone on total fat content in milk; therefore, the aforementioned mechanisms are not likely to cause the differences in MFG size.

Seeking a mechanism by which progesterone regulates MFG size, we assessed whether it affects the distribution of fatty acids between Tg and the membrane of MEC. Interestingly, the distribution of one of the major fatty acids in the cell, stearic acid, was greatly affected by the progesterone treatment. In cells treated with 1 ng/ml progesterone, stearic acid was found primarily in the membrane, whereas in cells treated with 5 ng/ml progesterone, it was mostly incorporated into the Tg. These results are most likely attributable to higher PL synthesis in the 1 ng/ml treatment, in accordance with the PL-quantification data (Figure 8). A higher content of PL may result in the formation of smaller lipid droplets, as smaller droplets require more surface material, provided by the PL (12, 19). Another interesting aspect of the PL in this study is their composition. PL composition can affect lipid droplet size by regulating its surface stability (12, 33–35), and hence fusion rates between adjacent droplets, which can contribute to their size. Because progesterone did not affect PL composition, the changes in lipid droplet size observed in the current study are most likely not attributable to fusion between droplets. Taken together, it can be concluded that the differences in lipid droplet size under different progesterone concentrations are caused by prioritized utilization of long-chain fatty acids for PL synthesis rather than Tg synthesis in MEC, without changing the total lipogenic capacity or the membrane composition.

One of the most prominent findings in the current study was that the differences in lipid droplet diameter and number were VLDL dependent. In the presence of VLDL, the proportion of MEC with small lipid droplets increased 2.4-fold when cultured with increasing progesterone concentration. In the absence of VLDL, no differences were found between cells incubated with increasing or decreasing progesterone levels. These findings clearly indicate that VLDL are involved in progesterone's mechanism of action. VLDL is one of the sources of long-chain fatty acids for MEC. Long-chain fatty acids are available for MEC from the circulation, either as non-esterified fatty acids originated from adipose lipolysis or from VLDL, the plasma vehicle responsible for the distribution of dietary fatty acids (36, 37). Long-chain fatty acids from VLDL are available to MEC either after endocytosis, executed by VLDL receptor, followed by lysosome hydrolysis, or by extracellular hydrolysis executed by membrane LPL (37). The LPL releases long-chain fatty acids near the basal side of the cell which are consequently taken up by CD36 (38). Availability of long-chain fatty acids can limit the synthesis capacity of membrane material and therefore affect lipid droplet size (19). In the mammary gland, LPL has been shown to be regulated by progesterone (21, 37). Although not examined in the current study, LPL seems to be the biochemical link between progesterone and lipid droplet size because differences in droplet size were only recorded when VLDL was included in the culture medium.

In summary, findings from both *in vivo* and *in vitro* models indicate that the pattern and direction (increasing vs. decreasing) of progesterone concentrations to which the MEC are exposed, rather than a specific progesterone concentration, affect MFG size. The findings extend our understanding of the mechanism underlying the regulation of MFG size and provide new evidence of a role for progesterone during lactogenesis.

## Data Availability Statement

All datasets generated for this study are included in the article/supplementary material.

## Ethics Statement

The animal study was reviewed and approved by Animal Experimentation Ethics Committee of the Agricultural Research Organization, The Volcani Center, Israel.

## Author Contributions

NA-A conceived and designed the experiments, wrote the article, and contributed reagents and equipment. CR designed and performed the experiments, analyzed the data, and helped write the article. ZR conceived, designed, and performed the experiments, analyzed the data, and wrote the article. All authors contributed to the article and approved the submitted version.

## Conflict of Interest

The authors declare that the research was conducted in the absence of any commercial or financial relationships that could be construed as a potential conflict of interest.
